# Antihypertensive Potential of *Pistacia lentiscus* var. *Chia*: Molecular Insights and Therapeutic Implications

**DOI:** 10.3390/nu16132152

**Published:** 2024-07-05

**Authors:** Panagiotis Efentakis, Lydia Symeonidi, Despoina D. Gianniou, Eleni V. Mikropoulou, Panagiota Giardoglou, Dimitrios Valakos, Giannis Vatsellas, Maria Tsota, Nikolaos Kostomitsopoulos, Ilias Smyrnioudis, Ioannis P. Trougakos, Maria Halabalaki, Georgios V. Dedoussis, Ioanna Andreadou

**Affiliations:** 1 Laboratory of Pharmacology, Faculty of Pharmacy, National and Kapodistrian University of Athens, Panepistimiopolis, 15771 Zografou, Greece; pefentakis@pharm.uoa.gr (P.E.); lydiasym98@hotmail.com (L.S.); 2 Department of Cell Biology and Biophysics, Faculty of Biology, National and Kapodistrian University of Athens, 15784 Athens, Greece; gndespoina@biol.uoa.gr (D.D.G.); itrougakos@biol.uoa.gr (I.P.T.); 3 Division of Pharmacognosy and Natural Products Chemistry, Department of Pharmacy, National and Kapodistrian University of Athens, 15771 Athens, Greece; e.mikropoulou@pharm.uoa.gr (E.V.M.); mariahal@pharm.uoa.gr (M.H.); 4 Department of Nutrition and Dietetics, School of Health Science and Education, Harokopio University of Athens, 17671 Athens, Greece; tota_giardoglou@yahoo.gr (P.G.); mtsota75@gmail.com (M.T.); dedousi@hua.gr (G.V.D.); 5 Center of Basic Research, Biomedical Research Foundation of the Academy of Athens, 11527 Athens, Greece; dvalakos@bioacademy.gr (D.V.); nkostom@bioacademy.gr (N.K.); 6 Greek Genome Centre, Biomedical Research Foundation of the Academy of Athens, 11527 Athens, Greece; gvatsellas@bioacademy.gr; 7 Mastiha Research Center, Seleperos Kallimasia, 82150 Chios, Greece; ismyrnioudis@gummastic.gr

**Keywords:** hypertension, chios mastic gum, phytotherapeutic, renal endothelium

## Abstract

**Background:** Hypertension poses a significant global health burden and is associated with cardiovascular morbidity. Chios mastic gum (CMG), derived from *Pistacia lentiscus* var. *Chia*, shows potential as a phytotherapeutic agent, due to its multifaceted beneficial effects. However, its anti-hypertensive effects and vascular, circulatory, and renal-related dysfunction, have not been thoroughly investigated. Herein, we aimed to explore the antihypertensive potential of CMG, focusing on vascular and renal endothelium, in vivo. **Methods:** Two models of hypertension in male rats, induced by Angiotensin II and Deoxycorticosterone acetate (DOCA)–high-salt administration, were utilized. CMG was administered at 220 mg/kg daily for four weeks after hypertension onset and blood pressure was measured non-invasively. Whole blood RNA sequencing, metabolomics, real-time PCR, and Western blot analyses of kidney and aorta tissues were additionally performed. **Results:** CMG significantly lowered systolic, diastolic, and mean blood pressure in both models. RNA sequencing revealed that CMG modulated immunity in the Angiotensin II model and metabolism in the DOCA–HS model. CMG downregulated genes related to oxidative stress and endothelial dysfunction and upregulated endothelial markers such as Vegfa. Metabolomic analysis indicated improved endothelial homeostasis via lysophosphatidylinositol upregulation. **Conclusions:** CMG emerges as a potent natural antihypertensive therapy, demonstrating beneficial effects on blood pressure and renal endothelial function.

## 1. Introduction

Hypertension stands as a prevailing global health challenge, raising critical concern in clinical practice and posing substantial risks to individuals’ cardiovascular health [1]. Its intricate interplay with various comorbidities, particularly regarding the cardiovascular system, underscores the urgency for innovative therapeutic approaches. Beyond its well-documented impact on myocardial function, hypertension exerts significant vascular and nephrological consequences, posing substantial risks to both the vascular and renal systems [2]. Persistently, elevated blood pressure leads to endothelial dysfunction, characterized by impaired vasodilation, increased vascular permeability, and enhanced oxidative stress, all of which contribute to the development of atherosclerosis and arterial stiffness [3]. This dysfunction initiates a detrimental cascade, promoting atherosclerosis, arterial stiffness, and ultimately, cardiovascular events. Mechanistically, hypertension-induced oxidative stress, inflammation, and dysregulated nitric oxide (NO) pathways contribute to endothelial dysfunction, exacerbating vascular compromise [4]. These vascular alterations not only heighten the risk of cardiovascular events, but also impair organ perfusion, exacerbating renal damage [2].

Hypertension-induced renal injury manifests through various mechanisms, including glomerular hypertension, oxidative stress, and inflammation, culminating in progressive renal dysfunction and the development of chronic kidney disease (CKD). Moreover, hypertension is a leading cause of hypertensive nephropathy, a specific form of CKD characterized by glomerular sclerosis, tubulointerstitial fibrosis, and eventual renal failure [5]. The complicated interplay between hypertension, vascular pathology, and renal dysfunction underscores the necessity for comprehensive management strategies, targeting both blood pressure control and organ-specific complications, to mitigate the considerable morbidity and mortality associated with hypertensive vascular and nephrological sequelae. 

*Pistacia lentiscus* var. *Chia*, commonly known as Chios mastic gum (CMG), has garnered considerable attention for its diverse pharmacological properties, including antioxidant, anti-inflammatory, vasodilatory, and cardioprotective effects [6,7]. CMG comprises of the natural polymer, namely *cis*-1,4-poly-β-myrcene, the essential oil which in turn is composed mostly of α-pinene and myrcene and the terpene fraction that can be further divided into the acidic and neutral terpenes. CMG’s most abundant compounds are the triterpenic acid isomers oleanonic, moronic, masticadienonic, and isomasticadienonic acids, with the last two being the most characteristic constituents of the resin [6]. Even though, to the best of our knowledge, CMG’s isolated triterpenes have not been thoroughly studied for their potential antihypertensive activity, several other triterpenic acids have stood out as potential antihypertensive agents, with the pentacyclic scaffold being more extensively investigated [8,9]. Throughout history, this plant has been employed in traditional medicine practices, across different cultures, for the management of various ailments, including cardiovascular disorders. Emerging preclinical and clinical scientific evidence has shed light on its potential as a novel therapeutic agent in the context of hypertension, offering a natural alternative to conventional pharmacotherapy. To date, the antihypertensive potential of long-term administration of chios mastic essential oil (CMEO) has been investigated in one clinical trial of hypertensive patients with cardiometabolic syndrome (CMS) [10] and one preclinical study. In the previous preclinical study of hypertensive rats, with an absence of cardiovascular confounders, undergoing renal artery stenosis by ligation of the renal artery for eight weeks, CMG was administered in the drinking water in a daily dose of 40 mg/kg for two weeks. CMG reduced both systolic and diastolic blood pressure, as well as renin and circulatory c-reactive protein (CRP) levels, and improved aortic elasticity in vivo. However, due to the minimal solubility of CMG in water [11], the actual per os administered dose lacks precision in the aforementioned in vivo model of hypertension. Additionally, the establishment of hypertension was surgically induced. Unilateral renal artery ligation, as therein performed, mimics the pathogenesis of renovascular hypertension observed in humans as a consequence of atherosclerotic renal stenosis [12]. Considering the nature of renovascular hypertension, which is classified as a secondary hypertension, closely related to ischemic renal disease [12], the translational value of the aforementioned study is limited. Finally, the exact mechanism of CMG’s antihypertensive potential was not investigated. 

In this study, we aim to unravel the effect of *Pistacia lentiscus* var. *Chia* on hypertension, shedding further light on its mechanism of action by focusing on circulation, vasculature, and the kidneys. We employed two translational in vivo models of hypertension, namely, the Angiotensin II (AngII) infusion via osmotic minipumps [13] and the Deoxycorticosterone acetate (DOCA)–high-salt (HS) administration [14], in male rats. These two models mimic the renin–angiotensin–aldosterone system (RAAS)-dependent and RAAS-independent primary hypertension observed in humans, respectively [15,16]. Since the antihypertensive potential of CMG has, so far, only been investigated in in vivo models of secondary hypertension [17], this illustrates the novelty of the present study. Subsequently, we investigated the regulation of signaling pathways in the circulation, the aorta, and the kidneys of the experimental animals receiving CMG, via RNA sequencing (RNAseq), metabolomics, and molecular analyses, seeking to identify molecular pathways related to its antihypertensive potential and focusing on the vascular and renal endothelium. The current work aims to identify and establish a translational rationale for the implementation of *Pistacia lentiscus* var. *Chia,* and, specifically, CMG, as an adjuvant antihypertensive phytotherapeutic, enabling its use in the clinical armamentarium of hypertension management. 

## 2. Materials and Methods

For complete materials and methods please refer to the Supplementary Materials online.

### 2.1. Animals

Twenty-four male Wistar rats, 12–14 weeks of age (300–350 g), were used for conducting this study. Experiments were performed in accordance with the “Guide for the care and use of laboratory animals” and experiments were approved by the Greek ethics committees (approval number: 179487/13-02-2023). Animals were housed and maintained in specific pathogen-free (SPF) cages (3/cage; 25 ± 1 °C) at least for one week before the experiments and had access to water and a normal chow diet ad libitum, according to ARRIVE guidelines [18]. Herein, only male rats were used for the conduction experiments. The use of male animals was selected, as male rats do not present the hormonal fluctuations that are due to the menstrual cycle observed in female rats and that lead to difficulties in data interpretation and increased variability of the results. However, according to sex relevance in preclinical models of hypertension [19], this is a limitation of the study and future studies should be conducted to investigate the sex differences in CMG antihypertensive potential.

### 2.2. Angiotensin II Model of Hypertension

Osmotic minipumps (#0000325 ALZET Model 2ML4, 2.5 μL/h, 4 weeks duration, Alzet^®^, Cupertino, CA, USA) were prefilled with 2 mg/mL AngII (#17150, human Angiotensin II, Cayman Chemical, Ann Arbor, MI, USA) in Normal Saline (N/S) corresponding to a dose of 0.4 mg/kg/day [13]. Minipumps were incubated in N/S for 24 h at 37 °C, prior to their implantation. For the establishment of the AngII-induced hypertensive model in rats, animals (n = 10) were anesthetized with isoflurane (1–2%). Subsequently the lower back of the rats was shaved, and a small incision was performed. A pocket was created subcutaneously and minipumps were implanted on the hinder part of the neck. Finally, skin was closed with 4.0 propylene sutures and rats were allowed to recover in their cages [13]. One week after the implantation of the minipumps, which is the timepoint of hypertension establishment [13], rats were divided into the following two groups: a control group, receiving N/S orally daily via gavage (n = 5) for 4 weeks; and a CMG group, receiving CMG (220 mg/kg) in N/S orally daily via gavage (n = 5) for 4 weeks. Due to low solubility of CMG in aqueous solutions [11], 2.8 g of CMG powder was suspended in 70 mL cold N/S and stored at 4 °C. CMG suspension was thoroughly vortexed prior to each administration to the rats. Fresh CMG suspension was prepared each week. At baseline, 1 week post-implantation, and 5 weeks post-implantation, rats underwent blood sampling and non-invasive blood pressure monitoring; at the endpoint of 5 weeks, rats were anesthetized by pentobarbital injection (50 mg/kg) and aorta and kidneys were excised and snap frozen in liquid nitrogen for further molecular analyses. 

### 2.3. DOCA-HS Model of Hypertension

DOCA was administered at a dose of 100 mg/kg, once weekly, subcutaneously to the rats. Additionally, rats were treated with 0.9% NaCl + 1% KCl via drinking water ad libitum [14]. At 3 weeks, which is the timepoint of hypertension establishment [14], rats were divided into the following two groups: a control Group, receiving N/S orally daily via gavage (n = 6) for 4 weeks; and a CMG group, receiving CMG (220 mg/kg) in N/S orally daily via gavage (n = 6) for 4 weeks. At baseline, 3 weeks post-treatment, and 7 weeks post-treatment, rats underwent blood sampling and non-invasive blood pressure monitoring; at the endpoint of 7 weeks, rats were anesthetized by pentobarbital injection (50 mg/kg) and aorta and kidneys were excised and snap frozen in liquid nitrogen for further molecular analyses.

### 2.4. Chios Mastic Gum Dose Selection

The dose selection of CMG was calculated based on the therapeutic dose, as proposed by the Chios Mastiha Growers Association, ranging from 1 to 5 g/person/day, and in compliance with data showing no severe adverse events of the natural product, up to the dose of 28 g/person/day [17]. Herein, since we challenged the antihypertensive potential of CMG after the establishment of hypertension, we used the highest therapeutic dose of the phytotherapeutic, that of 5 g/person/day, which corresponds to a rat-equivalent dose of 220 mg/kg according to dose extrapolation formulas [20].

### 2.5. Chios Mastic Gum Resin Analysis

CMG, collected in the “Kallimasia” area in Chios island and corresponding to the natural product administered to the rats, was analyzed by high-performance liquid chromatography/photodiode array detection/evaporative light scattering detector (HPLC-PDA-ELSD) (Agilent Technologies, Santa Clara, CA, USA) as previously described [21]. In brief, the powdered CMG was directly diluted in methanol (HPLC grade, Fisher Scientific, Leicestershire, UK), to achieve a final concentration of 5 mg/mL containing 100 μg/mL of internal standard (acetyl-ursolic acid, AUA). Samples were analyzed on an Agilent (Santa Clara, CA, USA) 1260 Infinity II HPLC, equipped with a 1260 Infinity II Quaternary Pump, a 1260 Infinity II Binary Pump and a 1260 Infinity II vial sampler, coupled linearly to a PDA and to a 1260 Infinity II ELSD. 

### 2.6. Blood Pressure Monitoring

For the measurement of arterial diastolic, systolic, and mean blood pressure, rats underwent non-invasive tail-cuff blood pressure assessment using the Coda Monitor System (Kent Scientific, Torrington, CT, USA) [22]. For the reliability of the measurements, rats were acclimated to the restrainers prior to the blood pressure monitoring, whereas 10 acclimation cycles were acquired prior to the actual measurements. Diastolic, systolic, and mean blood pressure per rat and per timepoint were calculated as an average of at least 10 correctly acquired measurement cycles.

### 2.7. Rat Plasma Liquid Chromatography—Mass Spectrometry Metabolomic Analysis

Plasma sample extraction was performed by protein precipitation and analysis by UPLC-ESI-Orbitrap-HRMS. Data processing was performed using the MzMine software version 2.53 [23] and compound annotation was based on HRMS and HRMS/MS spectral data comparison with online databases such as Human Metabolome Database (HMDB) [24], Metlin [25], and MassBank [26].

### 2.8. RNA-Sequencing Analysis

RNA was isolated from whole blood using Monarch^®^ Total RNA reagent (#T2010, New England Biolabs, Ipswich, MA, USA) as per manufacturer’s instructions. FASTQ format files were aligned to the Rattus norvegicus rn6 genome using HISAT2 program [27]. Counts were defined using the htseq-count command of HTSeq package v2.0.3 [28] using “intersection non-empty” mode and the “option” reverse regarding the library strandness, after removing the hemoglobin gene annotations from the rn6 gtf file. The count files were used as input for DESeq2 [29]. Differentially expressed genes (DEGs) were classified as those with a log2Fold-Change value less than −1.5 or greater than 1.5 and a *p*-value of ≤0.05. Pathway and Gene Ontology analysis was performed using the EnrichR web tool [30].

### 2.9. Real-Time PCR

For RNA isolation, kidneys and aortas were pulverized in liquid nitrogen and extracted using the standardized Trizol protocol. RT-PCR was performed with the CFX96 Real-Time PCR Detection System (Bio-Rad, Munich, Germany) using the SYBR^®^ Green method (Eva Green, Solis BioDyne, Tartu, Estonia) according to the manufacturer’s instructions [31].

### 2.10. Western Blot

Western blot analysis in aortic and kidney tissues was performed as previously described [32]. Primary antibodies against phospho-eNOS (Ser1177, #9571), eNOS (#32027), phos-pho-Akt (Ser473, #4060), Akt (#9272), and GAPDH (#2118) (Cell Signaling Technology, Europe, B.V., Eindhoven, The Netherlands) were used at 1:1000 dilution. Secondary HRP-linked antibodies, anti-mouse and anti-rabbit, were used (#7076, #7074 Cell Signaling Technology, Europe, B.V.) for protein visualization at 1:2000 dilution.

### 2.11. Statistical Analysis

Data are presented as means ± standard deviation (SD). Continuous variables were compared between two groups using parametric, unpaired Student’s *t*-test without assumption of consistent means. Multiple *t*-tests were used in the time-course assessment of CMG’s impact on arterial systolic, diastolic, and mean blood pressure. No assumption of equal variability of differences was performed and data were corrected with Greenhouse–Geisser correction. A *p*-value of at least <0.05 was considered statistically significant. All statistical analyses and graph preparation were performed using GraphPad Prism 8.5 analysis software (GraphPad Software, Inc., La Jolla, CA, USA) and SIMCA 17.0.2 analysis software. No outliers due to biological diversity were excluded. Samples that did not meet our technical criteria were not included in the analyses a priori. The absence of outlying values was confirmed by GraphPad Prism analysis software, using the ROUT method and Q = 1%. 

## 3. Results

### 3.1. Quantification of CMG Major Constituents

**Figure 1:**
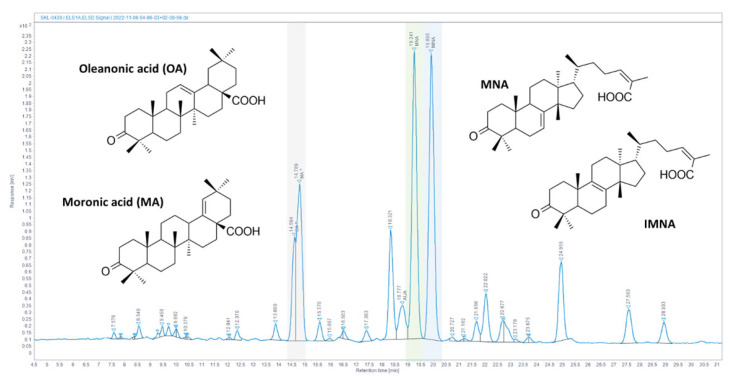
Chios mastic gum (CMG) resin analysis. HPLC-ELSD chromatogram of Chios mastic gum resin (CMG). Characteristic compounds are annotated. * Gray column corresponds to oleanonic acid (OA) and moronic acid (MA), green column corresponds to masticadienonic acid (MNA) whereas blue column corresponds to isomasticadienonic acid (IMNA).

The quantification of CMG’s major triterpenic acids was based on the calibration curves method using standards isolated in house [22]. Masticadienonic (MNA, PubChem CID: 44421209) and isomasticadienonic (IMNA, PubChem CID: 44421206) acid content was calculated at 17.4% and 15.5% (*w*/*w*) of crude material, respectively. The HPLC-ELSD chromatogram of CMG with major peaks annotated is presented in Figure 1.

### 3.2. CMG Reduced Systolic, Diastolic, and Mean Blood Pressure after Four Weeks of Administration Both in the AngII- and DOCA–HS-Induced In Vivo Models of Hypertension

**Figure 2:**
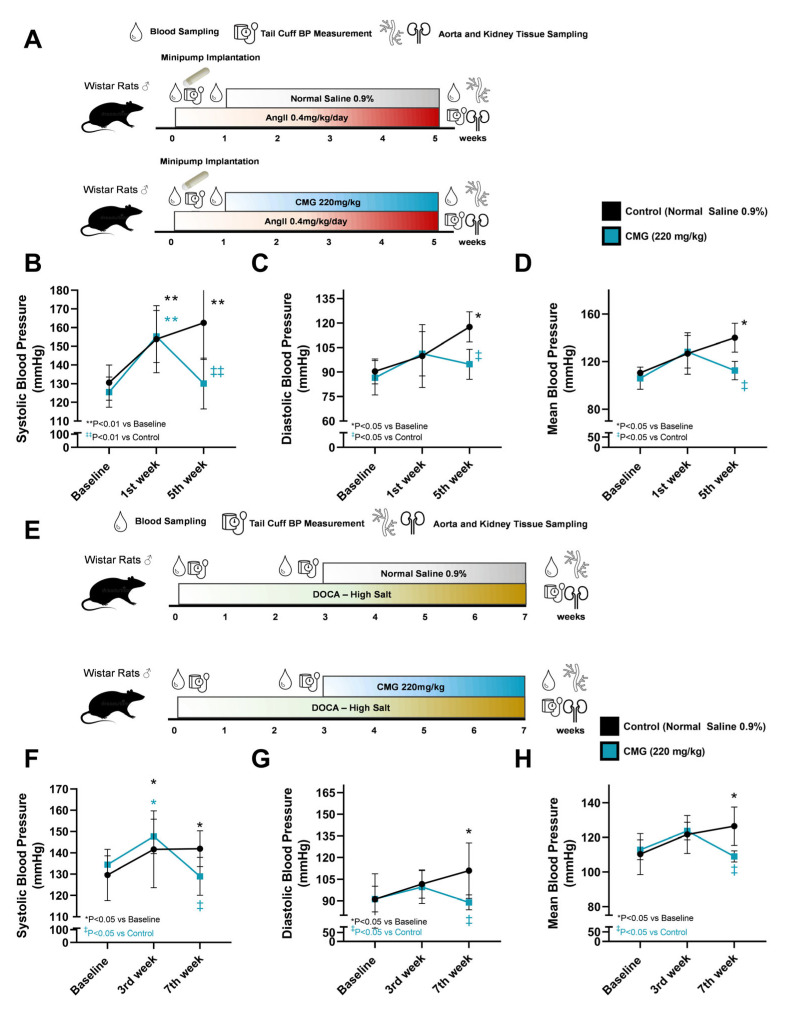
CMG exerts antihypertensive effect in both AngII- and DOCA–HS-induced hypertension in vivo. (**A**) Representative workflow of the AngII model in rats and graphs of (**B**) systolic, (**C**) diastolic, and (**D**) mean blood pressure (mmHg) (n = 5/group). (**E**) Representative workflow of the DOCA–HS model in rats and graphs of (**F**) systolic, (**G**) diastolic, and (**H**) mean blood pressure (mmHg) (n = 6/group). Data are presented as mean ± SD. Two-way ANOVA and Tukey’s post hoc analysis. AngII: angiotensin II, CMG: Chios mastic gum, DOCA: deoxycorticosterone acetate, HS: high salt.

Initially, we sought to investigate the antihypertensive potential of four weeks of CMG administration in vivo. Its antihypertensive potential was challenged in both AngII- and DOCA–HS-induced in vivo models of hypertension in rats, upon the establishment of hypertension. AngII infusion for one week led to a significant increase only in systolic blood pressure (153.8 ± 17.9 vs. 130.56 ± 9.4 at baseline in the control group and 155.2 ± 13.9 vs. 125.5 ± 8.1 at baseline in the CMG group, ** *p* < 0.01) in both the control and the CMG groups, whereas diastolic and mean blood pressure showed a tendency of increase, without reaching significance (Figure 2A–D). At five weeks, systolic (162.6 ± 19.6 vs. 130.56 ± 9.4 at baseline, ** *p* < 0.01), diastolic (117.6 ± 9.2 vs. 90.4 ± 7.6, * *p* < 0.05), and mean blood pressure (140.1 ± 12.2 vs. 110.5 ± 2.4 at baseline, * *p* < 0.05) were significantly increased only in the control group (Figure 2A–D). CMG exerted antihypertensive potential in the Ang-II model at five weeks, as it normalized systolic blood pressure compared to baseline and the control group (130.1 ± 13.6 vs. 125.5 ± 8.1 at baseline, *p* = ns and 130.1 ± 13.6 vs. 162.6 ± 19.6 in the control group, ^‡‡^ *p* < 0.01) and reduced systolic (94.8 ± 9.2 vs. 117.6 ± 9.2, in the control group, ^‡^ *p* < 0.05) and mean blood pressure (112.4 ± 7.7 vs. 140.1 ± 12.2, in the control group, ^‡^ *p* < 0.05) compared to the control (Figure 2A–D).

Regarding the DOCA–HS model, three weeks of DOCA–HS increased systolic blood pressure (141.7 ± 18.0 vs. 129.6 ± 12.0 at baseline in the control group and 147.7 ± 8.1 vs. 134.4 ± 4.1 at baseline in the CMG group, * *p* < 0.05), whereas no significant increase in diastolic and mean blood pressure was observed between the timepoints in any of the groups (Figure 2E–H), similarly to the AngII model. At seven weeks, systolic (141.9 ± 8.4 vs. 129.6 ± 12.0 at baseline, * *p* < 0.05), diastolic (111.0 ± 19.2 vs. 91.1 ± 9.8 at baseline, * *p* < 0.05), and mean (126.5 ± 3.2 vs. 110.4 ± 5.7) blood pressures were significantly increased only in the control group (Figure 2E–H). CMG retained its antihypertensive capacity in the DOCA–HS model, as it reduced systolic blood pressure (129.0 ± 8.9 vs. 134.4 ± 4.1 at baseline, *p* = ns and 129.0 ± 8.9 vs. 141.9 ± 8.4 in the control group, ^‡^ *p* < 0.05) and mitigated the increase in diastolic (89.0 ± 5.2 vs. 111.0 ± 19.2, ^‡^ *p* < 0.05) and mean blood pressure (109.0 ± 3.2 vs. 126.5 ± 11.1, ^‡^ *p* < 0.05) compared to the control (Figure 2E–H).

Collectively, four weeks of CMG administration, upon establishment of systolic hypertension, led to an antihypertensive effect after both AngII and DOCA–HS stimulation. Therefore, we sought to investigate its impact on the circulation, focusing, firstly, on circulatory gene expression in the whole blood.

### 3.3. CMG Led to Distinct Circulatory Gene Expression Profiles in the AngII- and DOCA–HS-Induced In Vivo Models of Hypertension. Emerging Role of Endothelial Homeostasis in CMG Antihypertensive Potential

**Figure 3:**
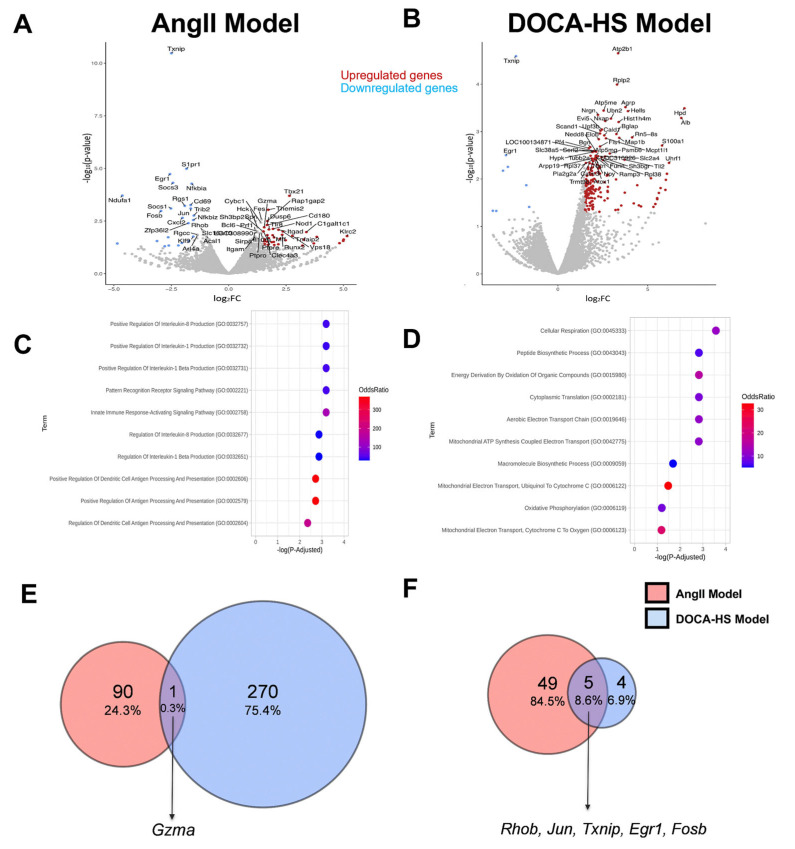
CMG leads to the upregulation of Gzma and downregulation of Rhob, Txnip, and Egr1 in the circulation in both in vivo models of hypertension. Volcano plots of differentially regulated expressed genes (DEGs) in the (**A**) AngII and (**B**) DOCA–HS in vivo models prior to and after CMG administration (red indicates the significantly upregulated genes and blue the significantly downregulated genes; cut-offs for Volcano plot: *p*-value < 0.05 and Log2FC > 1.5 and <−1.5). Gene Ontology analysis (Biological Process) regarding the upregulated genes derived from the (**C**) AngII and (**D**) DOCA–HS in vivo models. Venn diagram comparing the (**E**) upregulated genes and (**F**) downregulated genes derived from AngII (red) and DOCA–HS (blue) models. AngII: angiotensin II, CMG: Chios mastic gum, DOCA: deoxycorticosterone acetate, HS: high salt. Egr1: early growth response 1, Fosb: G0/G1 switch regulatory protein 3, Gzma: granzyme A, Jun: AP-1 transcription factor subunit, Rhob: Ras homolog gene family member B, Txnip: thioredoxin interacting protein.

In order to identify a putative molecular fingerprint of CMG in the circulation of the rats, in the two in vivo models of hypertension, we performed unbiased RNAseq analysis of the whole blood. Whole blood RNAseq analysis revealed a distinct set of upregulated genes in the AngII and DOCA–HS models after CMG administration (Figure 3A,B). Pathway and Gene Ontology analyses of the upregulated genes revealed that CMG administration significantly modulated immunity-related pathways, namely, the innate immune response-activating and pattern-recognition receptor signaling pathways and the production of positively regulated cytokines (Interleukin 1β, 1, and 8) in the AngII model. In the DOCA–HS model, CMG administration modulated the metabolic-related pathways, namely, cellular respiration, energy derivation by oxidation of organic compounds, peptide biosynthesis, aerobic electron transport chain, and mitochondrial respiration (Figure 3C,D). In order to identify the shared molecular targets of CMG in both models and the potential mediators of its antihypertensive capacity, we determined the common upregulated and downregulated genes in the two in vivo models. Vent diagrams of commonly differentiated genes revealed that CMG upregulated the granzyme A (Gzma) gene and downregulated the Ras homolog gene family member B (Rhob), AP-1 transcription factor subunit (Jun), thioredoxin-binding protein (Txnip), early growth response 1 (Egr1), and G0/G1 switch regulatory protein 3 (Fosb) genes (Figure 3E,F). Taking into account the aforementioned emerging role of metabolism in the antihypertensive potential of CMG, we subsequently performed LC-MS metabolomic analysis of the circulating metabolites of the experimental animals.

### 3.4. CMG Led to an Upregulation of Circulating Lysophosphatidylinositol Both in the AngII- and DOCA–HS-Induced In Vivo Models of Hypertension

**Figure 4:**
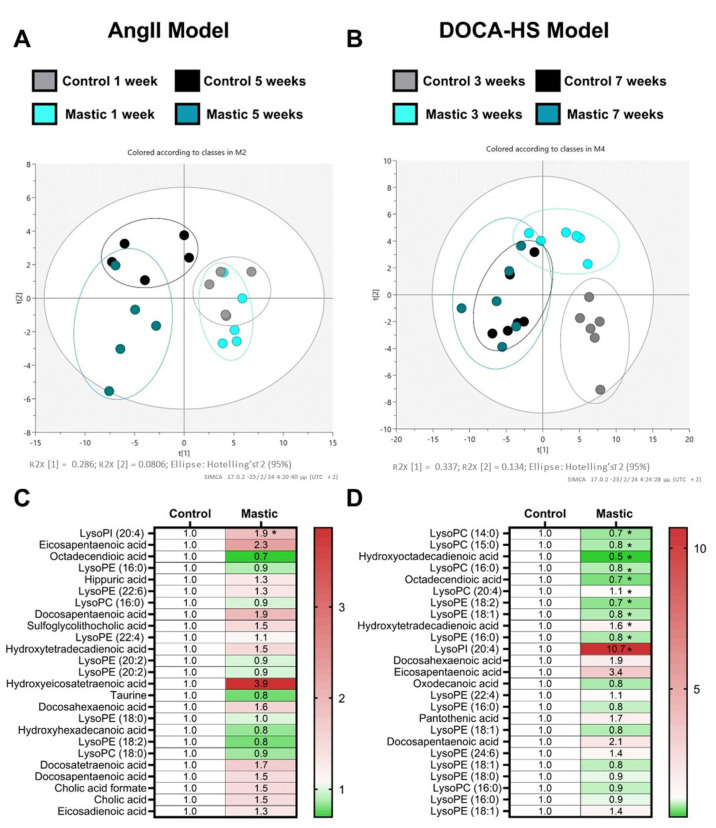
CMG increases lysophosphatidylinositol (20:4) in the circulation in both in vivo models of hypertension. Multivariate PLS-DA plots of identified circulatory metabolites in (**A**) the AngII (n = 5/group) and (**B**) the DOCA-HS (n = 6/group) in vivo models and heatmaps of identified metabolites in the (**C**) the AngII (n = 5/group) and (**D**) the DOCA-HS (n = 6/group). Unpaired, two-tailed *t*-test. * *p* < 0.05. CMG: Chios mastic gum, LysoPI: lysophosphatidylinositol, lysoPE: lysophosphatidylethanolamine, lysoPC: lysophosphatidylcholine.

Partial least squares discriminant analysis (PLS-DA) presented a distinct metabolic fingerprint between the time of hypertension establishment (one week and three weeks for the AngII and DOCA–HS models, respectively) and the endpoint of the in vivo experiments (five weeks and seven weeks for the AngII and DOCA–HS models, respectively) (Figure 4A,B). LC-MS metabolomic analysis of the circulating metabolites revealed a distinct metabolic profile of CMG in the AngII model of hypertension, as the samples exhibit separative clustering at the t2 principal component (Figure 4A). On the contrary, CMG did not present a definite metabolic shift in the DOCA–HS model (Figure 4B). Metabolites assessment showed that CMG led to a significant upregulation of lysophosphatidylinositol (LysoPI) (20:4) in the AngII model (Figure 4C); whereas it led to a significant downregulation of lysophosphatidylcholines (LysoPC) (14:0), (15:0), (16:0), and (20:4); lysophosphatidylethanolamine (LysoPE) (18:2), (18:1), and (16:0); and hydroxyoctadecadienoic and hydroxytetradecadienoic acids. A significant CMG-induced upregulation of LysoPI (20:4) was observed in DOCA–HS model (Figure 4D), in line with the AngII model. Conclusively, it seems that upregulation of LysoPI (20:4) emerges as a ubiquitous metabolic fingerprint of CMG in both in vivo models of hypertension. 

### 3.5. CMG Exerted an Endothelium-Mediated Protection Effect in the Hypertensive Kidney and Aorta In Vivo

**Figure 5:**
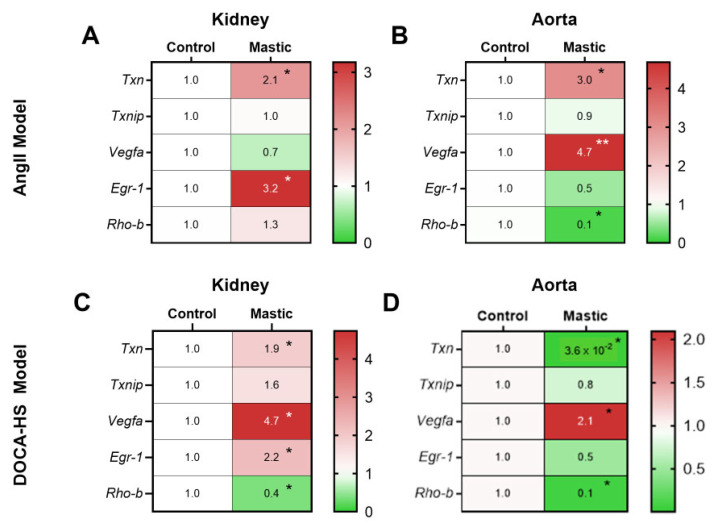
CMG increased antioxidant and endothelial targeted genes in the kidneys and aortas of the AngII- and DOCA–HS-induced in vivo models of hypertension. Heatmaps of Txn, Txnip, Vegfa, Egr1, and Rhob genes in the (**A**) kidney and (**B**) aorta of the AngII-treated rats. Heatmaps of Txn, Txnip, Vegfa, Egr1, and Rhob genes in the (**C**) kidney and (**D**) aorta, and graphs of the DOCA–HS-treated rats. Unpaired, two-tailed *t*-test. * *p* < 0.05, ** *p* < 0.01. AngII: angiotensin II, CMG: Chios mastic gum, DOCA: deoxycorticosterone acetate, Egr1: Early growth response 1, HS: high salt, Rhob: Ras homolog gene family member B, Txn: thioredoxin, Txnip: thioredoxin interacting protein, Vegfa: vascular endothelial growth factor A.

Since we identified multiple potential targets of CMG’s antihypertensive potential in the circulation, we subsequently sought to explore the regulation of the aforementioned targets in the key organs for blood pressure regulation, namely, the kidneys and the aorta [33]. RT-PCR analysis showed a significant upregulation of thioredoxin (Txn) mRNA expression both in the kidneys and the aorta of the AngII-infused rats at the endpoint and an upregulation of Egr1 in the kidneys and the vascular endothelial growth factor A (Vegfa) mRNA expression in the aortas in the CMG group. Rhob mRNA expression was found to be decreased only in the aortas of the CMG -treated rats in the AngII model (Figure 5A,B). Additionally, an upregulation of Vegfa mRNA expression in the kidneys and the aortas and an increase in Txn gene expression was observed only in the kidneys of the CMG-treated animals in the DOCA–HS model (Figure 5C,D). Taking into account that TXN contributes to oxidative stress mitigation and antioxidant endothelial defense [34], alongside with the protective potential of both Vegfa and Egr1 in the microvascular endothelium [35,36], we further focused our mechanistic studies on the CMG-induced endothelial regulation in the selected organs.

### 3.6. CMG Increases Renal Endothelial NO Synthase Phosphorylation Both in the AngII- and DOCA-HS-Induced In Vivo Models of Hypertension

**Figure 6:**
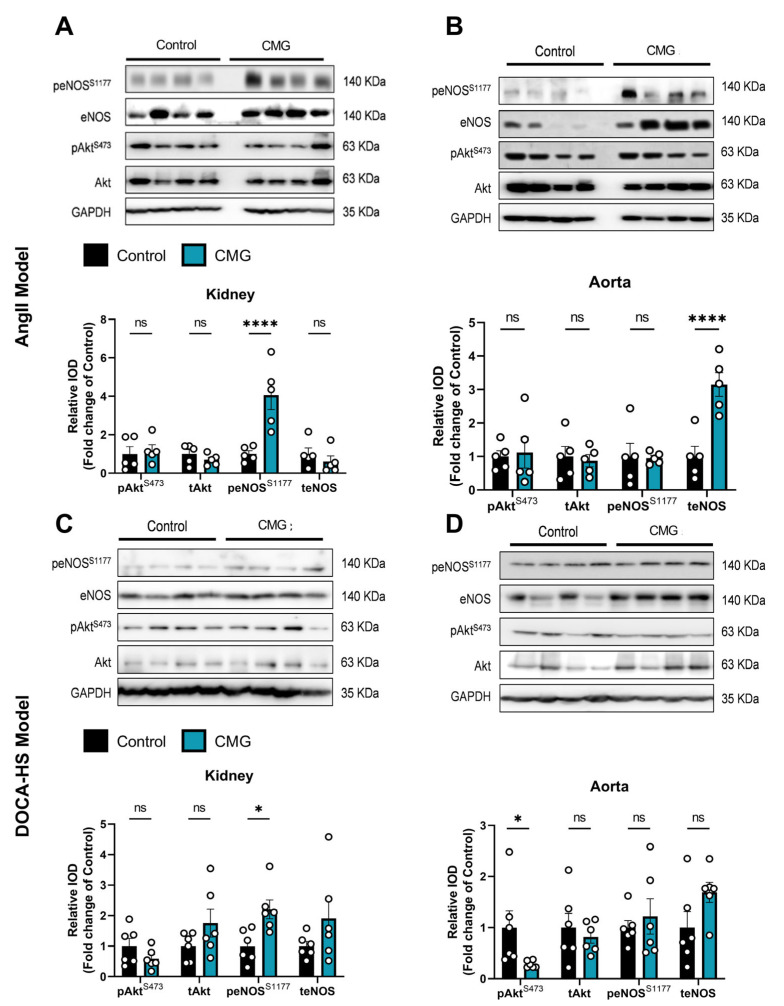
CMG leads to renal eNOS phosphorylation in both in vivo models of hypertension. Representative Western blot images and relative densitometry analysis of phospho-Akt and phospho-eNOS in the (**A**) kidney and (**B**) aorta of the AngII-treated rats (n = 5/group) and (**C**) kidney and (**D**) aorta of the DOCA–HS treated rats (n = 6/group). Data are presented as mean ± SD. Unpaired, two-tailed *t*-test. * *p* < 0.05, **** *p* < 0.001, ns: not significant. Akt: protein kinase B, AngII: angiotensin II, CMG: Chios mastic gum, DOCA: deoxycorticosterone acetate, HS: high salt, eNOS: endothelial nitric oxide synthase.

Following the emerging role of the endothelium in the hypertensive potential of CMG, we sought to investigate the protein kinase B (Akt)-endothelial NO synthase (eNOS) pathway in the kidneys and aortas of the animals. We found that in the AngII model, CMG administration led to an increased phosphorylation of eNOS in the kidneys (Figure 6A) and upregulation of eNOS expression in the aorta (Figure 6B), without any effect on Akt regulation in any of the selected tissues. Additionally, we observed that CMG led to a significant increase in eNOS phosphorylation in the DOCA–HS-induced hypertensive kidneys (Figure 6C), whereas only a decrease in Akt phosphorylation in the aortas was observed in the same in vivo model (Figure 6D). Consecutively, CMG seems to favorably induce eNOS phosphorylation in the kidneys, possibly improving the renal microvascular regulation of blood pressure in vivo.

## 4. Discussion

Hypertension is the leading cause of worldwide cardiovascular morbidity and mortality. Hypertension management, through lifestyle changes and medication, remains of paramount importance in reducing the risk of cardiovascular events and improving overall health outcomes [37]. Herein, we challenged the antihypertensive potential of CMG after the establishment of hypertension in vivo, seeking to decipher its mechanism of blood pressure regulation.

Osmotic minipump implantation with AngII in rodent models of hypertension holds significant translational value in cardiovascular research. Exploiting the chronic infusion of AngII, this in vivo model enables the simulation of hypertensive conditions in rats, facilitating the study of disease mechanisms and therapeutic interventions. Such models provide insights into the pathophysiology of hypertension, including vascular remodeling, renal dysfunction, central nervous system (CNS) activation and cardiac hypertrophy, illustrating key aspects of the essential primary hypertension in humans [16]. The activation of RAAS and the vast production of ROS seem to be the main pathomechanisms of AngII-induced hypertension in the aforementioned model [15,38]. The second in vivo model, the DOCA–HS model, possesses additional translational value, as the pathomechanism of DOCA–HS simulates low-renin hypertension, resistant to angiotensin converting enzyme inhibitors (ACEIs) and angiotensin receptor blockers (ARBs), in humans [15]. Taking into consideration that drugs targeting RAAS in the latter patients lead to suboptimal hypertension management and high cardiovascular morbidity and mortality risk, new therapeutic approaches are required for treating these high-risk individuals [39]. Our results showed that four-week CMG administration successfully mitigated hypertension in both in vivo models, by lowering systolic, diastolic, and mean blood pressure. Taking into consideration that DOCA–HS-induced hypertension is proposed to be RAAS-independent and relies primarily on CNS- and renal-mediated phenomena [40], we could propose that CMG’s antihypertensive potential is mediated through a mechanism divergent from RAAS inhibition.

The circulatory system, comprising the myocardium, blood vessels, and blood, plays a vital role in maintaining whole-body homeostasis [41]. Therefore, we initially investigated the impact of CMG on gene regulation in the whole blood via unbiased RNA-seq. We found divergent antihypertensive fingerprints of CMG in the two in vivo models. In the AngII model, in which innate immunity plays a key role in the establishment of vascular inflammation and hypertension [42], CMG differentially regulated the innate immune response-activating and pattern-recognition receptor signaling pathways. Innate and adaptive immunity are key players in the induction of hypertension. Immunodeficient mice are found to be protected against hypertensive complications, such as endothelial dysfunction, oxidative stress, increased vascular tone, renal interstitial infiltrates, sodium retention, and kidney injury [43]. Therefore, the immune regulatory potential of CMG, in the circulation may contribute to its antihypertensive potential. 

Concerning the DOCA–HS model, RNA-seq analysis revealed that CMG could positively regulate mitochondrial-related pathways, such as cellular respiration and aerobic electron transport chain. Since metabolism emerged as the key pathway fingerprint of CMG in the aforementioned model, and metabolism is also a pivotal mediator of immune responses [44], we performed a LC-MS metabolomic analysis in both in vivo models, in the plasma. We found that CMG led to a consistent upregulation of LysoPI in both models. LysoPI is a bioactive lipid generated by the phospholipase A (PLA) family of lipases which is believed to play a significant role in several physiological and pathological processes including cell growth, differentiation, and motility; in a wide range of cell-types including cancer cells, endothelial, and nervous cells; acting, among others, as a mitogenic factor [45]. Improving mitochondrial health is emerging as a novel feasible approach to treat hypertension [46]. Since hypertension-mediated mitochondrial dysfunction results in impaired energy production, deficient calcium regulation, decreased activity of anti-oxidant enzymes, and the oxidative modification of cellular compartments, which subsequently leads to end-organ damage in the heart, the brain, the kidneys, and the vasculature [47], the CMG-induced upregulation of the mitogenic LysoPI might contribute to the antihypertensive mechanism of CMG. Moreover, the LysoPI implication in improved mitochondrial dynamics in endothelial cells [45] might point towards an endothelial targeted mechanism of action of CMG. 

Since our multiomic analyses revealed multiple pathways involved in the circulation, we sought to identify the shared gene targets of CMG in the two in vivo models. Vent chart analysis identified one shared upregulated gene, namely *Gzma* and five shared downregulated genes, namely *Rhob*, *Jun*, *Txnip*, *Egr1*, and *Fosb*. Among the identified shared targets, Rhob is known to be a proapoptotic molecule leading to endothelial cell death in terms of vascular biology and pathology [48]. Txnip, an endogenous inhibitor of the antioxidant protein TXN, is a notorious mediator of endothelial dysfunction and hypertension [49]; Egr1, which has been previously shown to play a renoprotective role in acute kidney injury [50] and is identified as a marker of neointimal remodeling, correlates to progression in human arterial hypertension [51]. Therefore, all aforementioned targets’ downregulation might be implicated in the antihypertensive potential of CMG, pointing towards an endothelial-targeted mechanism of action. Therefore, we subsequently focused on investigating these targets’ regulation and the expression patterns of their related downstream effectors, i.e., *Txn* and *Vegfa*, in the kidneys and aortas of the experimental animals. 

Our RT-PCR analysis deduced an upregulation of *TXN* both in the aortas and the kidneys in the CMG-treated rats in the AngII cohort. TXN is a key antioxidant enzyme suppressing the serum-free-induced hydroxyl radicals, lipid peroxidation, and apoptosis [52,53]. The downregulation of *Txnip* in the circulation and *TXN* in the renal and aortic tissues might comprise an enhanced multi-faceted antioxidant defense mechanism induced by CMG. Interestingly, the upregulation of TXN has been shown to confer endothelial antioxidant capacity in the apolipoprotein E-deficient mouse and improve NO bioavailability, supporting the endothelial-targeted effect of CMG [34]. Additionally, we found that in the AngII-treated rats, CMG upregulated *Egr1* in the kidneys and *Vegfa* in the aortas. It has previously been shown that Egr1 enhances kidney repair by activating SRY-box transcription factor 9^+^ (SOX9^+^) in renal tubular cells [50], whereas EGR-1 serves as a transcriptional factor for Vegfa [54]. On the other hand, Vegfa leads to endothelial cell proliferation, migration, and survival in the vessels, exerting vasoprotection [55]. Importantly, the association of Vegf inhibition with hypertension is well recognized, since anticancer drugs acting as Vegf inhibitors lead to hypertensive complication, as their primary vascular adverse effect [56]. In the DOCA–HS model, RT-PCR analysis in the aortic and renal tissues showed an upregulation of *Txn*, *Vegfa*, and *Egr1* in the kidneys, whereas only an upregulation of *Vegfa* was observed in the aortas of the CMG treated rats, in line with the AngII model. Taking into account the consistent upregulation of *Egr1* and *Vegfa*, our RT-PCR data add on the endothelial-targeting potential of CMG. The former hypothesis is additionally reinforced by the downregulation of *Rhob* mRNA in the AngII-treated aorta and DOCA–HS-treated kidney and aorta, possibly indicating an endothelial-targeted anti-apoptotic potential of CMG [57]. 

Taking together the above results indicating that CMG targets endothelial function, we further investigated the activation of the eNOS pathway in the renal and aortic tissues of our in vivo models. NO produced by eNOS is a fundamental determinant of cardiovascular homeostasis as it regulates systemic blood pressure, vascular remodeling, and angiogenesis [58]. Numerous preclinical studies indicate that natural compounds [59,60,61,62] and pharmacological agents [63,64,65] mitigate hypertension via the activation of the eNOS pathway in the endothelium. Herein, we found that CMG increased eNOS phosphorylation in the kidneys of both AngII- and DOCA-HS-treated rats. Downregulation of eNOS in the renal medulla is shown to induce hypertension in rats [66], whereas significant constitutive expression of NO synthases in the juxtaglomerular apparatus of the kidneys are negative regulators of renin secretion and thereupon RAAS system activation [67]. It is, therefore, well established that activation of renal eNOS is a therapeutic modality for managing hypertension. CMG has been consistently found, within our study, to increase renal eNOS activation, potentially leading to improved renal microvascular endothelial cell homeostasis and contributing to its antihypertensive potential. 

In a recent randomized controlled trial, focusing on individuals with abdominal obesity and metabolic abnormalities, Chios mastic essential oil (CMEO) demonstrated significant therapeutic potential [10]. Interestingly, a reduction in systolic blood pressure and alanine aminotransferase levels was observed in CMEO-treated patients. The dose of 200 mg of CMEO used in the aforementioned study translates to a total dose of approximately 6 g of CMG per patient. Taking into account that our translational dose in rats corresponds to 5 g per patient, the dose regimen selected in both studies is similar. Although, endothelial function serves as a shared mediator in the intricate interplay between cardiovascular metabolic confounders and hypertension [68], no mechanistic data were provided in the aforementioned clinical trial, rendering our study innovative regarding the antihypertensive potential of CMG and its mechanism of action.

## 5. Conclusions

Conclusively, we have shown, in vivo, that long-term CMG administration at a translational therapeutic dose can exert antihypertensive potential, in both AngII-induced and DOCA–HS-mediated hypertension. Our multiomic analysis revealed multiple targets on CMG’s action in the circulation, with improved endothelial homeostasis emerging as the converging outcome of CMG administration in both models. Further molecular analyses showed that CMG favorably induces endothelial protective effects on the renal endothelium, standing as a novel mechanism of the antihypertensive potential of CMG. Since hypertension remains the leading cause of cardiovascular morbidity and mortality in the general population, new means of decreasing hypertension prevalence are necessary for the improvement of cardiovascular health. Phytotherapeutics, such as CMG, that possess a safe toxicology profile might serve as new adjuvant antihypertensive remedies in the clinical practice armamentarium.

## Data Availability

Raw and processed data are uploaded at the Gene Expression Omnibus datasets database with GSE265763 as accession number. The data remain in private status and reviewers can access the record with the following secure token: ylyfsmiupbwnvmb.

## Supplementary Materials

The following supporting information can be downloaded at: https://www.mdpi.com/article/10.3390/nu16132152/s1, Table S1: Real-Time PCR primers used for target validation in kidneys and aortas. Real-Time PCR primers sequence, melting temperature and product size, used for target validation in kidneys and aortas in the AngII- and DOCA-HS in vivo models of hypertension. Txn, Thioredoxin; Txnip, Thioredoxin interacting protein; Egr-1, Early growth response protein 1; Vegf-a, Vascular endothelial growth factor A.
