# Supplementary material for: Antihypertensive Potential of Pistacia lentiscus var. Chia: Molecular Insights and Therapeutic Implications
Source: Nutrients. 2024 Jul 5;16(13):2152. doi: 10.3390/nu16132152 (PMC11243328; doi:10.3390/nu16132152)
Supplement: Supplementary file 1 [file nutrients-16-02152-s001.zip › nutrients-3061094-supplementary.pdf]

nutrients

MDPI

Article

Antihypertensive potential of Pistacia lentiscus var. Chia: Molecular Insights and Therapeutic Implications.

Panagiotis Efentakis[1], Lydia Symeonidi[1], Despoina D. Gianniou[2], Eleni V. Mikropoulou[3], Panagiota Giardoglou[4], Dimitrios Valakos[5], Giannis Vatsellas[6], Maria Tsota[4], Nikolaos Kostomitsopoulos[5], Ilias Smyrnioudis[7], Ioannis P. Trougakos[2], Maria Halabalaki[3], Georgios V. Dedoussis[4], Ioanna Andreadou[1#].

[1] Laboratory of Pharmacology, Faculty of Pharmacy, National and Kapodistrian University of Athens, Athens, Greece.
[2] Department of Cell Biology and Biophysics, Faculty of Biology, National and Kapodistrian University of Athens, Athens, Greece.
[3] Division of Pharmacognosy and Natural Products Chemistry, Department of Pharmacy, National and Kapodistrian University of Athens, Athens, Greece.
[4] Department of Nutrition and Dietetics, School of Health Science and Education, Harokopio University of Athens, Athens, Greece.
[5] Center of Basic Research, Biomedical Research Foundation of the Academy of Athens, Athens, Greece.
[6] Greek Genome Centre, Biomedical Research Foundation of the Academy of Athens
[7] Mastiha Research Center, Seleperos Kallimasia, Chios, Greece

* **Corresponding author:** Ioanna Andreadou, Laboratory of Pharmacology, Faculty of Pharmacy, Panepistimiopolis, Zografou, Athens 15771, Greece; tel: +30 210 7274827; fax: +30 210 7274747; e-mail: jandread@pharm.uoa.gr

Abstract: Background: Hypertension poses a significant global health burden and is associated with cardiovascular morbidity. Chios mastic gum (CMG), derived from *Pistacia lentiscus var. Chia*, shows potential as a phytotherapeutic agent, due to its multifaceted beneficial effects. However, its anti-hypertensive effects and vascular, circulatory, and renal-related dysfunction, have not been thoroughly investigated. Herein, we aimed to explore the antihypertensive potential of CMG, focusing on vascular and renal endothelium, in vivo. **Methods:** Two models of hypertension in male rats by Angiotensin II and Deoxycorticosterone acetate (DOCA)-High Salt administration, were utilized. CMG was administered at 220 mg/kg daily for four weeks after hypertension onset and blood pressure was measured non-invasively. Whole blood RNA sequencing, metabolomics, real-time PCR, and Western blot analyses of kidney and aorta tissues were additionally performed. **Results:** CMG significantly lowered systolic, diastolic, and mean blood pressure in both models. RNA sequencing revealed that CMG modulated immunity in the Angiotensin II model and metabolism in the DOCA-HS model. CMG downregulated oxidative stress- and endothelial dysfunction-related genes and upregulated endothelial markers such as Vegfa. Metabolomic analysis indicated improved endothelial homeostasis via lysophosphatidylinositol upregulation. **Conclusion:** CMG emerges as a potent natural antihypertensive therapy, demonstrating beneficial effects on blood pressure and renal endothelial function.

Keywords: hypertension; chios mastic gum; phytotherapeutic; renal endothelium

1. Supplemental material and methods

1.1. Rat plasma liquid chromatography – mass spectrometry metabolomics

Plasma sample extraction was performed by protein precipitation. 100 μL of plasma were extracted by vortexing with 800 μL of ice-cold (−20°C) acetonitrile (ACN) [liquid chromatography-mass spectrospcopy (LC-MS) grade, Merk, Darmstadt, Germany]. Following centrifugation at 4°C, 12000 x rpm for 10 min (Mikro 200 R centrifuge, Andreas Hettich GmbH, Tuttlingen Germany), the supernatants were dried in an Eppendorf (Hamburg, Germany) vacufuge concentrator with no heating applied. Samples were reconstituted in 200 μL of the mobile phase prior to analysis by LC-MS. A pooled plasma sample was prepared by mixing equal volumes of all study samples prior to extraction.

LC-MS analysis was carried out on an Acquity H-Class Ultra Performance Liquid Chromatography (UPLC) System (Waters, Millford, MA, USA) coupled to a Thermo Velos Pro Orbitrap Elite (Thermo Scientific, Watham, MA, USA) hybrid mass spectrometer equipped with a heated electrospray ionization source. Separation was achieved on an Acquity HSS T3 (Waters) column (100 × 2.1 mm, 1.8 μm) using a gradient consisting of water (MilliporeSigma, Burlington, MA, USA) with 0.1% (*v*/*v*) formic acid (LC-MS grade, Merck) (A) and ACN (B). Elution started at 2% B, which was maintained for 2 min and increased to 100% B for another 16 min. These conditions were maintained for 2 min before returning to the initial conditions in 1 min for a 4-min re-equilibration (25 min in total). Column temperature was set at 40°C and the flow rate was set to 0.4 mL/min. Source heater and capillary temperatures were set to 350 °C and the source voltage was 2.7 kV. Rf lens level was tuned at 45%. 5 μL of sample were injected into the system. High-resolution mass spectrometry (HRMS) data were acquired in the negative ionization mode in the full scan *m*/*z* range of 113 – 1000 with a resolution of 60000. Data-dependent acquisition was simultaneously performed using three dependent scan events, with a CID value of 35% and a mass resolution of 30000. Nitrogen was used as the sheath gas (45 au) and auxiliary gas (15 au). Spectral acquisition was performed using the XCalibur software version 2.2.

Data processing was performed using the MzMine software version 2.53 [1]. A standard workflow was applied including steps such as peak picking, chromatogram deconvolution, deisotoping and alignment, peak filtering was performed by removing features present in blank solvent samples. Compound annotation was based on HRMS and HRMS/MS spectral data comparison with online databases such as Human Metabolome Database (HMDB) [2], Metlin [3] and MassBank [4].

1.2. RNA-sequencing analysis

RNA was isolated from whole blood using Monarch® Total RNA reagent (#T2010, New England Biolabs, Ipswich, Massachusetts, USA) as per manufacturer's instructions. RNASeq experiments were carried out in the Greek Genome Center (GGC) of the Biomedical Research Foundation of the Academy of Athens (BRFAA). RNASeq libraries were prepared with the NEBNext Ultra II Directional RNA Library Prep Kit for Illumina, with 1 μg of total RNA input. Library quality control (QC) was performed with the Agilent bioanalyzer DNA1000 kit and quantitation with the qubit spectrophotometric method. Sequencing was performed at Illumina NovaSeq 6000 system, generating at least 20 million 101 bp Single-End reads for each sample. QC was performed on the FASTQ raw data file for each sample using the FASTQC software. FASTQ files were aligned to the *Rattus norvegicus* rn6 genome using HISAT2 [5]. Counts were defined using the htseq-count command of HTSeq package v2.0.3 [6] using "intersection non-empty" mode and the "option" reverse regarding the library strandness, after removing the hemoglobin gene annotations from the rn6 gtf file. The count files were used as input for DESeq2 [7]. Normalization was performed with the estimate size factor function followed by Differentially Expressed Genes (DEGs) analysis. As DEGs were classified those with a log2Fold-Change value less

than −1.5 or greater than 1.5 and a p-value of ≤0.05. Pathway and Gene Ontology analysis was performed using the EnrichR web-tool [8].

1.3. Real-Time PCR

For RNA isolation, kidneys and aortas were pulverized in liquid nitrogen and extracted by the standardized Trizol protocol. RT-PCR was performed with the CFX96 Real-Time PCR Detection System (Bio-Rad, Munich, Germany). Isolated RNA was reverse-transcribed to cDNA using high-capacity cDNA reverse transcription kit (FastGene Scriptase II cDNA, Nippon Genetics, Japan). Specific primer pairs (Eurofins Genomics AT, GmbH) were designed (Primer-Blast, NCBI, NIH) and used in order to detect mRNA expression of target genes, using the SYBR® Green method (Eva Green, Solis BioDyne, Estonia) according to the manufacturer's instructions [9]. Real-time PCR primers' sequences, melting temperatures (Tms) and product sizes are presented in **Table S1**.

Table S1: Real-Time PCR primers used for target validation in kidneys and aortas.

Table S1			
Gene name	Primers (F/R)	Tms	Product Size
Txn (*Rattus Norvergicus*)	AGCTGATCGAGAGCAAGGAAG	59.86	83
	CGTGGCAGAGAAGTCCACTA	59.11	
Txnip (*Rattus Norvergicus*)	ACCAGCCTACAGGTGAGAAC	59.02	80
	AGGAAGCTCAAAGCCGAACT	59.60	
Egr-1 *Rattus Norvergicus*)	GCACCTGACCACAGAGTCCTTT	62.45	89
	ACCGGGTAGTTTGGCTGGG	61.90	
Vegf-a *Rattus Norvergicus*)	GCACATAGGAGAGATGAGCTTCC	60.55	99
	CACCGCCTTGGCTTGTCACAT	63.78	

Real-Time PCR primers sequence, melting temperature and product size, used for target validation in kidneys and aortas in the AngII- and DOCA-HS in vivo models of hypertension. Txn, Thioredoxin; Txnip, Thioredoxin interacting protein; Egr-1, Early growth response protein 1; Vegf-a, Vascular endothelial growth factor A.

1.4. Western Blot

Western Blot analysis in aortic and kidney tissues was performed as previously described [10]. Snap-frozen tissue samples were lysed in lysis buffer containing 1% Triton-X, 20mM tris(hydroxymethyl)aminomethane (Tris-Base) (adjusted pH 7.4), 150mM NaCl, 50mM KF, 1 mM ethylenediaminetetraacetic acid (EDTA), 1mM ethylene glycol-bis(β-aminoethyl ether)-N,N,N',N'-tetra-acetic acid (EGTA), 1% sodium dodecyl sulphate (SDS), 0.5% sodium deoxychollate and 0.1% protease/phosphatase inhibitor cocktail, and homogenized. Lysates were centrifuged for 15 min (13000 x g, 4°C). Supernatants were used for the determination of total protein by the Lowry method and samples preparation with Dave's buffer (4% SDS, 10% 2-mercaptoethanol, 20% glycerol, 0.004% bromophenyl blue, and 0.125 M Tris–HCl), as previously described [10]. An equal amount of protein was loaded into each well, separated by SDS-PAGE and transferred onto a polyvinylidene difluoride membrane (PVDF), which was blocked for 1-2 hours with 5% non-fat dry milk.

Membranes were incubated overnight at 4°C with primary antibodies and for 2 hours with secondary horseradish peroxidase (HRP)-conjugated antibodies at room temperature. Probed membrane signals were detected using chemiluminescent HRP substrate (Luminata Forte Western HRP substrate, Millipore, USA) and imaged using an automated cooled CCD imager (ImageQuant LAS 500, GE Healthcare Bio-Sciences, Sweden). Relative densitometry analysis was conducted using ImageJ 1.49v software (National Institutes of Health, USA).

Primary antibodies against phospho-eNOS (Ser1177, #9571), eNOS (#32027), phospho-Akt (Ser473, #4060), Akt (#9272) and GAPDH (#2118) (Cell Signaling Technology, Europe, B.V.) were used at 1:1000 dilution. Secondary HRP-linked antibodies anti-mouse and anti-rabbit were used (#7076, #7074 Cell Signaling Technology, Europe, B.V.) for protein visualization at 1:2000 dilution.

2. References

1. Pluskal, T.; Castillo, S.; Villar-Briones, A.; Oresic, M. MZmine 2: modular framework for processing, visualizing, and analyzing mass spectrometry-based molecular profile data. *BMC Bioinformatics* **2010**, *11*, 395, doi:10.1186/1471-2105-11-395.
2. Wishart, D.S.; Guo, A.; Oler, E.; Wang, F.; Anjum, A.; Peters, H.; Dizon, R.; Sayeeda, Z.; Tian, S.; Lee, B.L., et al. HMDB 5.0: the Human Metabolome Database for 2022. *Nucleic Acids Res* **2022**, *50*, D622-D631, doi:10.1093/nar/gkab1062.
3. Guijas, C.; Montenegro-Burke, J.R.; Domingo-Almenara, X.; Palermo, A.; Warth, B.; Hermann, G.; Koellensperger, G.; Huan, T.; Uritboonthai, W.; Aisporna, A.E., et al. METLIN: A Technology Platform for Identifying Knowns and Unknowns. *Anal Chem* **2018**, *90*, 3156-3164, doi:10.1021/acs.analchem.7b04424.
4. Horai, H.; Arita, M.; Kanaya, S.; Nihei, Y.; Ikeda, T.; Suwa, K.; Ojima, Y.; Tanaka, K.; Tanaka, S.; Aoshima, K., et al. MassBank: a public repository for sharing mass spectral data for life sciences. *J Mass Spectrom* **2010**, *45*, 703-714, doi:10.1002/jms.1777.
5. Kim, D.; Langmead, B.; Salzberg, S.L. HISAT: a fast spliced aligner with low memory requirements. *Nat Methods* **2015**, *12*, 357-360, doi:10.1038/nmeth.3317.
6. Anders, S.; Pyl, P.T.; Huber, W. HTSeq--a Python framework to work with high-throughput sequencing data. *Bioinformatics* **2015**, *31*, 166-169, doi:10.1093/bioinformatics/btu638.
7. Love, M.I.; Huber, W.; Anders, S. Moderated estimation of fold change and dispersion for RNA-seq data with DESeq2. *Genome Biol* **2014**, *15*, 550, doi:10.1186/s13059-014-0550-8.
8. Kuleshov, M.V.; Jones, M.R.; Rouillard, A.D.; Fernandez, N.F.; Duan, Q.; Wang, Z.; Koplev, S.; Jenkins, S.L.; Jagodnik, K.M.; Lachmann, A., et al. Enrichr: a comprehensive gene set enrichment analysis web server 2016 update. *Nucleic Acids Res* **2016**, *44*, W90-97, doi:10.1093/nar/gkw377.
9. Efentakis, P.; Lamprou, S.; Makridakis, M.; Barla, I.; Nikolaou, P.E.; Christodoulou, A.; Dimitriou, C.; Kostomitsopoulos, N.; Ntanasis-Stathopoulos, I.; Theochari, I., et al.

Mineralocorticoid Receptor Pathway Is a Key Mediator of Carfilzomib-induced Nephrotoxicity: Preventive Role of Eplerenone. *Hemasphere* **2022**, *6*, e791, doi:10.1097/HS9.0000000000000791.

10. Efentakis, P.; Kremastiotis, G.; Varela, A.; Nikolaou, P.E.; Papanagnou, E.D.; Davos, C.H.; Tsoumani, M.; Agrogiannis, G.; Konstantinidou, A.; Kastritis, E., et al. Molecular mechanisms of carfilzomib-induced cardiotoxicity in mice and the emerging cardioprotective role of metformin. *Blood* **2019**, *133*, 710-723, doi:10.1182/blood-2018-06-858415.
